# Detector response and dose calculation lateral to material interfaces for 6 MV photon beam

**DOI:** 10.1002/acm2.70302

**Published:** 2025-10-21

**Authors:** Jonas Ringholz, Otto Andreas Sauer, Sonja Wegener

**Affiliations:** ^1^ Department of Radiation Oncology University Hospital Würzburg Würzburg Germany

**Keywords:** detector response, dose calculation, inhomogeneities

## Abstract

**Background:**

Dose calculation around inhomogeneities is challenging for many algorithms. A validation of dose distributions in these conditions is not straightforward, as detector response close to material interfaces is affected by the non‐equilibrium situation, the changing energy spectrum and the volume effect in a steep dose gradient.

**Purpose:**

Detector response lateral to an inhomogeneity of high density was studied, mimicking the situation of bone surrounded by soft tissue.

**Methods:**

Profiles obtained with different detectors (diodes, ion chamber, synthetic diamond) in water in the vicinity of an aluminum cylinder were compared. Dose deposition was also calculated with different commercial treatment planning systems and algorithms as well as using Monte Carlo simulations within and lateral to the cylinder and compared to measurements.

**Results:**

Dose deposition in the vicinity of an inserted aluminum cylinder changes and is registered by the detectors to a different degree. The ion chamber shows the largest change irradiated with a 10×10 cm^2^ at 3 mm distance from the cylinder surface (2.5%), followed by the synthetic diamond (1.7%), then the unshielded (1.4%) and finally the shielded diode (1.0%). Dose calculated by different commercial dose engines differed up to 3% at that point from the detector values (Collapsed Cone).

**Conclusions:**

Dose calculation near inhomogeneities depends on the used algorithm, dose measurements in the same region differ depending on the detector type used. We recommend verification of dose calculation with second type of algorithm and measurements with at least two detector types.

## 1 INTRODUCTION

Dose accuracy within about three to five percent is expected from a radiotherapy treatment.^1^, ^2^ Multiple factors contribute to deviations between the planned and the deposited dose, including absolute and relative dose measurements, beam modeling, dose calculation, linear accelerator (linac) performance, patient positioning and patient‐related factors. The accuracy of dose calculation depends on the algorithms used for this purpose. While correct calculation in homogeneous media is easily accomplished by algorithms, dose calculation around material inhomogeneities has been repeatedly reported to be erroneous.^3^, ^4^, ^5^, ^6^, ^7^, ^8^, ^9^

Historically, the first treatment planning software tools would parameterize dose distributions measured in water phantoms as a function of source to surface distance, field size, depth, and lateral position. Non‐water‐equivalence of the patients’ tissue was considered by applying factors, which considered the differences in attenuation.^10^ Since calculation speed has increased, the early approaches started to be replaced by model‐based algorithms in the 1980s.^11^, ^12^, ^13^ The main concept of these algorithms is calculating the energy released to the medium by interactions of the primary photons, convoluting this with the kernels that describe how the dose by scattering photons and electrons is deposited around the primary photon interaction site.^10^, ^11^, ^12^ Model‐based algorithms rely on many approximations. While fairly accurate for many situations, limitations exist, especially around lung tissue^6^, ^8^, ^14^ and bone.^5^, ^8^

A third class of algorithms directly acknowledges the underlying physical processes. Monte Carlo algorithms models are a stochastic method to simulate transport and dose deposition of particles and implemented in many commercial planning systems today.^15^, ^16^ Radiation transport can also be described by Boltzmann transport equations, and the Acuros XB algorithm directly solves these.^17^ However, calculation algorithms implemented in state‐of‐the‐art planning systems include simplifications to achieve reasonable calculation times.^18^, ^19^

In addition to the inherent limitations of the used algorithms, treatment planning in inhomogeneous media is further complicated by missing guidance on how to verify calculated dose against measurements. Recommended steps in beam commissioning validation are typically performed in simple slab phantoms for simple field geometries.^20^, ^21^ These cases are an oversimplification of the highly complex anatomical structures encountered in treatment planning, for example within the lung or the head‐and‐neck region. Exemplary dose measurements in anthropomorphic phantoms are recommended upon commissioning^20^ but the majority of plan validation is done in homogeneous phantoms, such as the common quality assurance phantoms of different geometries consisting of plastic materials such as polysterol or polymethylmethacrylat (PMMA).

From the current perspective, it is not feasible to demand more measurements in complex anatomical shapes or including inhomogeneous media without being able to recommend a detector for those measurement tasks. Detector response in non‐equilibrium situations is challenging. Small field dosimetry is one situation of missing charged particle equilibrium, which has been extensively studied in the literature^22^, ^23^ albeit mainly limited to the central axis of small fields in homogeneous media.

Studies on detector response near material inhomogeneities often concentrate on changes to depth dose curves.^7^, ^8^, ^24^ There is little data on detector response at the interface between the water phantom surface and the surrounding air in the build‐up region. Correction factors for detector response within an anthropomorphic phantom have been derived by Monte Carlo simulation for a Farmer chamber.^25^, ^26^ What remains largely unstudied is the detector response lateral of material inhomogeneities.^27^, ^28^ The detector response for this measurement task is influenced by the volume effect due to the steep profile, energy‐dependence of the response due to the changing energy spectrum out of field, and detector composition due to the asymmetry of the task at hand.

This work aims to provide systematic data on the detector response close to material interfaces, both for a large field exceeding the size of the inhomogeneity and for a small field irradiating only a part of the inhomogeneity and scattering radiation to the surrounding medium. Different commonly used detectors were studied in the vicinity of an aluminum cylinder in a water phantom, which served as a simple model for the anatomical situation of bone surrounded by normal tissue. Moreover, dose calculations in the same geometry were performed with different dose engines and Monte Carlo simulations.

## 2 MATERIAL AND METHODS

### 2.1 Measurements

#### 2.1.1 Phantom setup

**FIGURE 1:**
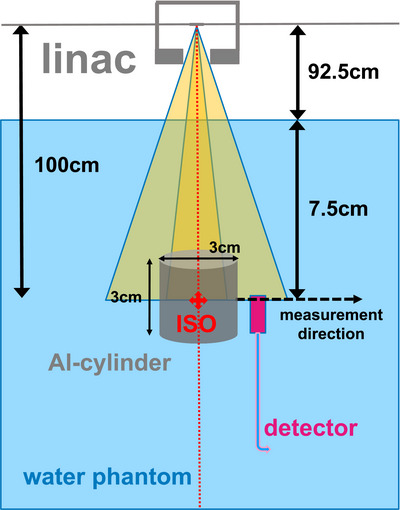
Setup of the aluminum cylinder in the water phantom.

An aluminum cylinder (aluminum alloy, density: 2.8 g/cm^3^), 3 cm in diameter as well as in height, was inserted as an inhomogeneity into a PTW MP3‐XS water phantom (PTW‐Freiburg, Freiburg, Germany), placed axially in a 3D‐printed mounting (Figure 1). The cylinder center was positioned in the linac isocenter by optical guidance of the lasers. The source to surface distance was set as 92.5 cm, leaving 6 cm of water above the cylinder top. After alignment of the field axis and the cylinder axis, the setup was irradiated with a 6 MV photon beam using an Elekta Synergy linac (Elekta, Stockholm, Sweden) from 0° gantry angle. Two fields were irradiated, a nominal 10×10 cm^2^ field exceeding the cylinder size, and a nominal 2×2 cm^2^ field (effective field size calculated as the geometric mean: *s*_eff _= (2.032 ± 0.034) cm) irradiating only cylinder material.

Studied detectors were mounted in the phantom holder consecutively. Detector signals were obtained at different positions around the cylinder. Radial measurements were recorded at a depth of 7.5 cm starting from as close to the lateral cylinder surface as detector dimensions allowed, moving the detector radially outwards. MEPHYSTO mc^2^ dosimetry software with the control unit of the Tandem electrometer (both PTW‐Freiburg) for the detector movement in the water phantom was used for detector positioning. Measurements were performed with a UNIDOS electrometer (PTW‐Freiburg). A spatial resolution of 0.5 mm up to 25 mm to the beam center and 1 mm at larger distances was set. 400 monitor units (MU) were irradiated per measurement point. Each measurement was repeated in a homogenous water phantom at the same measurement positions as before and, additionally, in the field center. For these measurements, the cylinder was moved to a corner at the bottom of the phantom to keep the water level constant for all measurements.

All studied detectors are listed with their specifications in Table 1. Before the measurement, detectors were aligned to the setup using the CenterCheck function of MEPHYSTO mc^2^. For the depth adjustment, diodes were positioned with their effective measurement point as indicated by the manufacturer and ion chambers were positioned with their central axis at water surface level, which was defined as a depth of 0.

**TABLE 1:** Properties of detectors used in this study.

Name	Type	Orientation	*r*_d_ (mm)	*r*_eff_ (mm) axial/radial
microDiamond (60019)	synthetic diamond	axial	1.1	1.0/
Diode 60016	shielded diode	axial	0.56	1.2/
Diode 60012	unshielded diode	axial/radial	0.56	0.6/
PinPoint 3D (31022)	ion chamber	radial	1.45	/2.4
EBT3	radiochromic film	orthogonal to beam	pixel size 0.17 mm (150 dots per inch scan resolution)	0.14 mm (film center)

*r*_d_: Radius of detector area according to the recommended mounting direction. *r*_eff_: Distance from detector surface to effective measurement point of detector. All detectors were manufactured by PTW Freiburg (Freiburg, Germany).

#### 2.1.2 Solid state detectors

For the study we used a microDiamond detector (PTW 60019, PTW‐Freiburg) and two p‐type diodes: an unshielded (PTW 60012, PTW‐Freiburg) and a shielded diode (PTW 60016, PTW‐Freiburg). All three diodes are designed for axial measurements. Nevertheless, we also performed measurements in radial orientation with the PTW 60012, the detector with the smallest distance between the outer front surface and the indicated measurement point, minimizing the gap between the cylinder surface and the first radial measurement point. No biasing voltage was applied to the detectors and the detectors were pre‐irradiated as recommended by the manufacturers.

#### 2.1.3 Ionization Chamber

The PinPoint 3D Ion Chamber (PTW 31022 PTW‐Freiburg) is a compact cylindrical chamber. A biasing voltage of 400 V was applied to the chamber and it was pre‐irradiated according to the manufacturer's specification for each session. The ion chamber was used only in the radial orientation.

#### 2.1.4 Radiochromic film

For film dosimetry EBT3 Gafchromic film (Ashland Advanced Materials, Bridgewater, NJ, USA) was used. Film strips were mounted between two halves of the aluminum cylinder extending approximately 5 cm into water along the measurement path used for radial detector measurements. Otherwise, the setup remained unchanged. For both field sizes, the strips were irradiated with 200 monitor units (MU). The measurements were repeated without the cylinder. All measurements were performed three times per configuration and field size.

Calibration measurements were performed in water iso‐centrically at a depth of 10 cm irradiating nine film strips to known doses ranging from 0 to 10 Gy. The film strips were digitized with an Epson 11000XL scanner (Epson, Suwa, Japan). Optical density was converted into dose using Film QA Pro (Ashland, Covington, USA), using the three color channel technic^29^ together with the one scan protocol.^30^ All details followed the procedure described previously.^31^, ^32^

#### 2.1.5 MLC leakage measurement for the 2×2cm^2^ field

Measurements were performed in an RW3 Slab Phantom (PTW‐Freiburg). One slab with a detector‐specific hole was used together with a PTW Semiflex 3D chamber (PTW‐Freiburg, Freiburg, Germany) at a depth of 7.5 cm, the same depth as used in the aluminum cylinder setup. The multileaf collimator (MLC) leakage was determined by measuring the profiles in in‐plane and cross‐plane direction through a 2×2 cm^2^ field D_2x2_ extending approximately 5 cm from the field edge and through a field with closed MLC *D*_closed_. The phantom setup as a whole, including the inserted detector, was moved to different positions with respect to the radiation field by moving the linac couch. Comparing the two profile measurements out of field, the ratio *d*_leak_ was determined from the signals in the closed field *S*_closed_ and in the open field S_2x2_ at a radial position of 4 cm as

(1) $$d_{\mathrm{leak}}=S_{\mathrm{closed}}/S_{2\times 2}.$$

### 2.2 Implementation in the treatment planning software

Computed tomography (CT) imaging of the water phantom setup including the aluminum cylinder was acquired using a SOMATOM go.Open Pro scanner (Siemens Healthineers, Erlangen, Germany) in order to model the exact measurement geometry. The CT images were imported into two treatment planning systems, Pinnacle (Philips Radiation Oncology Systems, Milpitas, CA, USA, version 16.2.1) and Eclipse (Varian Medical Systems, Palo Alto, USA, version 18.0).

Based on the phantom dimensions in the CT, a virtual phantom was constructed in each treatment planning system assigning the physical material parameters to components as required: For Pinnacle, 1.0 g/cm^3^ and 2.7 g/cm^3^ mass density was assigned to water and aluminum, respectively, and for Eclipse, materials water and aluminum were assigned to the structures. For these materials Eclipse used the same values as Pinnacle as default densities. Air density (0.0012 g/cm^3^ and 0.00125 g/cm^3^ in Pinnacle and Eclipse, respectively) was assigned to the surrounding medium. The dose was calculated with a spatial resolution of 1 mm (Pinnacle, both fields, Eclipse 2 × 2 cm^2^‐field) or 2 mm (Eclipse, 10 × 10 cm^2^‐field) due to limitations in computational capacities of the used hardware setup.

### 2.3 Monte Carlo setup

The program‐package EGSnrc^33^ was used for simulations of the measurement setup. To save computing time, we used the sub‐package DOSRZnrc^34^ for a radial symmetric geometry. All relevant details can be found in Table 2.

**TABLE 2:** MC simulation parameters reported according to the AAPM Task Group Report 268.^35^

**Checklist item #**	**Item name**	**Description**	**References**
2,3	Code, version/release date	EGSnrc (2020), DOSRZnrc (2020)	^33^, ^34^
4, 17	Validation	Code, including the radial geometry, has been used in similar studies before.	^36^
5	Timing	Total simulation time approximately 300 hours with an Intel® Xeon(R) CPU E5‐1680 v3 @ 3.20 GHz × 16.	
8	Source description	Source 0: Photons, parallel circular beams of 2.2 cm and 11.16 cm diameter, monoenergetic with 17 distinct energies between 0.1 MeV and 8 MeV	
9	Cross‐sections	Photonen: xcom‐library, Brems: Bethe Heitler (BH)	^37^
10	Transport parameters	Charged particles are transported ‐ Pair angular sampling: simple ‐ Brems angular sampling: Koch‐Motz 2BS (KM) ‐ Brems cross sections: Bethe‐Heitler (BH) ‐ Electron‐step algorithm: PRESTA‐II ‐ Electron impact ionization: off ‐ Boundary crossing algorithm: EXACT: Single scatter mode ‐ Global ECUT: 0.521MeV ‐ Global PCUT: 0.01MeV ‐ Global SMAX 1e10cm ‐ Rayleigh scattering: On ‐ Bound Compton: norje ‐ PE angular sampling: On ‐ Atomic relaxations: On ‐ ESTEPE: 0.25 ‐ Xlmax: 0.5 ‐ Skin depth for BCA 3 ‐ Spin effects: On	^34^ ^38^
11	VRT and/or AEIT	No Variance Reduction Techniques were used.	
12	Scored quantities	Dose to medium (i.e. either dose to aluminum, water or the respective detector material depending on the simulation geometry)	
13,18	# histories/statistical uncertainty	160 million histories per geometry	
14	Statistical methods	Statistical uncertainties obtained directly from simulation results.	
15,16	Postprocessing	No filtering of the MC results performed. Doses were calculated from simulation results by summation according to linac spectra including inter leaf leakage, as detailed in section 2.3. For calculation of ratios, see equation 2.	

**FIGURE 2:**
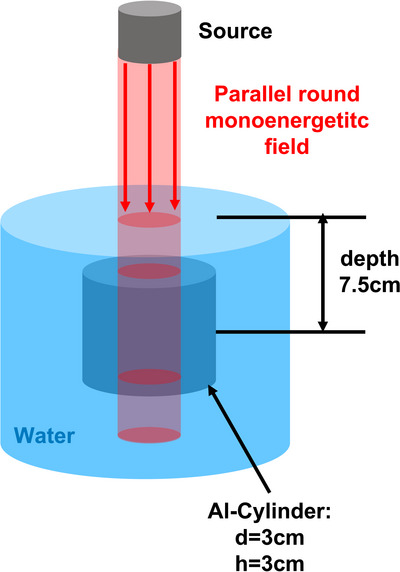
Setup of the Monte Carlo implementation in the package DOSrz of EGSnrc.

A cylindrical water phantom (Figure 2) was defined with a diameter of 30 cm, placing the aluminum cylinder on the central axis in the same depth as in the measurement setup. Radiation fields were parallel circular beams with field sizes chosen as the circular equivalent field to the square fields used in our measurement setup, yielding diameters of 2.2 cm and 11.16 cm according to our previous equivalent square study.^31^ The smaller field size was chosen to represent field sizes typically found in intensity‐modulated radiotherapy treatment techniques. The model was simulated for 17 monoenergetic photon energies between 0.1 MeV and 8 MeV. The monoenergetic energy results were weighted with an ELEKTA Synergy 6 MV spectrum.^39^ The simulations were also repeated without the aluminum cylinder in the phantom. For each simulation 160 million histories were computed. The dose in the aluminum cylinder was calculated as dose to aluminum. The relevant radial dose to medium was calculated in a 1 mm thick radial layer from inside the cylinder to the outer phantom part.

For a better comparison to the film measurement, we performed also two simulations modeling the film measurement in more detail. As a first approximation, a 0.3 mm thick slab composed of water was inserted between the two cylinder halves to calculate the film dose. As a final model, we inserted the film layers as specified by the manufacturer^40^ and recorded the dose from the active layer as dose to medium.

Similarly we performed also simulations for the two types of detectors we used for the measurement. For each detector a radial detector position of 18 mm was simulated. For the simulations the detector volumes were defined in DOSrz for cylindrical symmetric reasons in such a way that the detector volumes were extended circularly around the aluminum cylinder at a radial distance of 18 mm. The PinPoint 3D chamber was created with an air chamber of 2.9 mm in diameter surrounded by a 0.09 mm thick graphite shell which is surrounded by a 0.57 mm PMMA layer. As diode we choose the PTW‐60012. For the simulation the detector shell was simplified assumed to be built of water. For the detection part of the diode we used a 0.5 mm thick and in radial direction 1.65 mm long silicon slab. As detection volume only a 2.5 µm thick and 1.12 mm long layer on the top and middle of the silicon slab was used.

To take also the inter leaf leakage and transmission radiation for the smaller field size into account, the simulated dose grid for the larger field was superimposed with a factor of *d*_leak _= 0.04 onto the doses of the smaller field size. The factor *d*_leak_ was determined as described in section 2.1.4.

### 2.4 Data Analysis

#### 2.4.1 Film Analysis

The film dose matrix was exported in order to analyze it using an in‐house developed Python^41^ (Version 3.6.9) tool.

To average the three measurements for each setup, different approaches for the 2×2 cm^2^ and the 10×10 cm^2^ field measurement were implemented. For the 2×2 cm^2^ setup, the whole field was captured with the film strips. Therefore, the field center could be detected based on the region with maximum dose and the fields were aligned accordingly. This region was identified by employing an algorithm using a square window with a width of 140 pixels to scan the dose grid for the highest mean dose.

For the 10×10 cm^2^ field measurements, only one field edge was recorded on the film strip. Here, the dose matrices of the measurements with aluminum cylinder in the field were matched at the cylinder edge by minimizing the mean‐dose difference between the films in the selected gradient region. The edge of the radiation field was used to mutually align the measurements without the cylinder and to those with a cylinder present.

Finally, a radial dose profile was created as the dose average over eleven pixels (each approximately 0.16 mm side lengths) orthogonal to the radial axis, that is five neighboring pixels to each side of the profile.

#### 2.4.2 Data processing and normalization of the dose measurements

All data was normalized to the signal $s_{\mathrm{water},10}\left(0\right)$ obtained centrally in the 10×10 cm^2^ field for the respective detector. This yields a normalized signal

(2) $$S_{\mathrm{setup},\mathrm{FS}}\;\left(x\right)=\frac{s_{\mathrm{setup},\mathrm{FS}}\left(x\right)}{s_{\mathrm{water},10}\left(0\right)}\;,$$

in which $s_{\mathrm{setup},\mathrm{FS}}\left(x\right)$ is the measured signal of a given detector at a radial distance x from the field center of a field of field size *FS* (indicated by either 10 for the 10×10 cm^2^ or 2 for the 2×2 cm^2^ field), in the respective setup, which can be either $s_{\mathrm{water},\mathrm{FS}}$ in water or $s_{\mathrm{cylinder},\mathrm{FS}}$ with the cylinder present.

For each detector the ratio $r_{FS}\left(x\right)$ at the lateral position x from the cylinder center was calculated from signals $S_{\mathrm{cylinder},\mathrm{FS}}\left(x\right)$ measured with and from signals $S_{\mathrm{water},\mathrm{FS}}\left(x\right)$ measured without the aluminum cylinder in the phantom at the same lateral position and indicated field size *FS*:

(3) $$r_{\mathrm{FS}}\left(x\right)=\frac{S_{\mathrm{cylinder},\mathrm{FS}}\left(x\right)}{S_{\mathrm{water},\mathrm{FS}}\left(x\right)}\;.$$

For profiles from the film, the TPS and MC simulations dsose instead of signals were entered into Equation 2.

#### 2.4.3 Volume averaging effect

The volume averaging occurs especially in fields, where the field size reaches the size of the detector volume or in regions with a steep dose‐gradient where the averaging of the dose in the detector volume leads to a significant reduction or increase of the dose compared to the actual dose on the reference point of the detector. In this study especially the regions of the field edge of the 2×2 cm^2^ field and the region near the aluminum cylinder for the 10×10 cm^2^ field are of interest. To calculate the effect the measurement data of the PTW‐60012 diode in radial orientation was fitted in these regions with an exponential function with additional linear and parabolic terms. Afterwards around every measurement point the function was integrated over the one dimensional detection dimensions and was divided by the detection diameter.

#### 2.4.4 Uncertainties

Detector signal reproducibility was obtained from repeated measurements. Reproducibility / noise uncertainties for film measurement were estimated using the standard deviation of the three measurement strips and the averaging of 11 pixels orthogonal to the dose profile as explained above. Uncertainties for Monte Carlo simulations were obtained from the simulation results.

Uncertainties regarding measurement and detector setup, material composition and TPS dose grid were obtained from varying parameters in the Eclipse treatment planning system. Recalculated and original doses were compared at two positions 17 mm and 25 mm away from the field center.

## 3 RESULTS

### 3.1 Measurements

**FIGURE 3:**
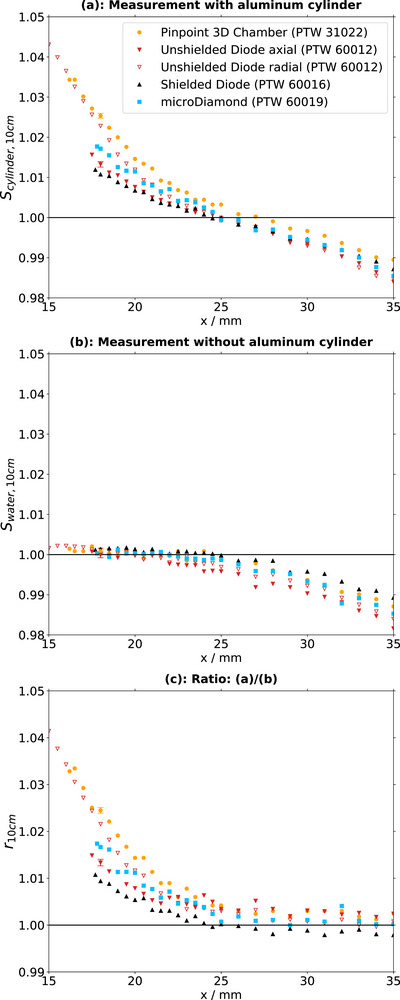
Data for all 10 × 10 cm^2^‐field measurements. (a): Profiles with the cylinder in the field. (b): Profiles without the cylinder in the field. (c): Ratios of profiles with to without the cylinder included in the field.

**FIGURE 4:**
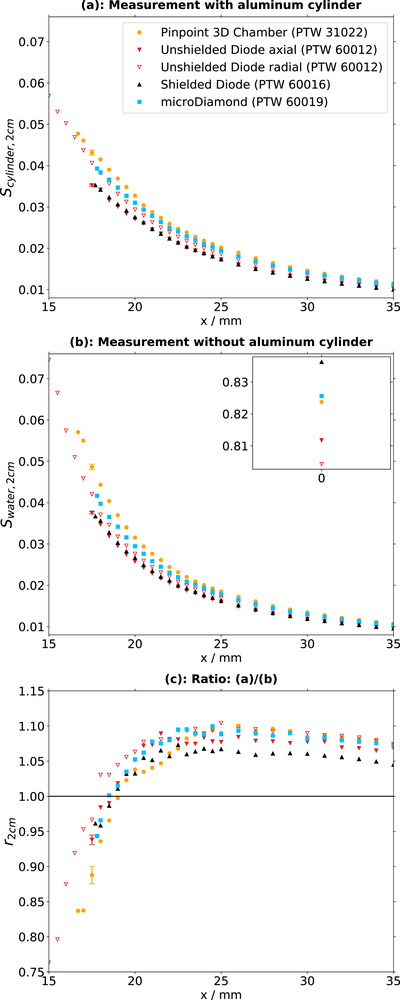
Data for all 2 × 2 cm^2^‐field measurements. (a): Profiles with the cylinder in the field. (b): Profiles without the cylinder in the field. (c): Ratios of profiles with to without the cylinder included in the field.

Measured lateral profiles are displayed both with the cylinder in place (Figures 3a and 4a) and in a solely water‐filled phantom without the cylinder present (Figure 3b and 4b). It depends on the detector geometry (Table 1) how close each detector can approach the cylinder edge at position x = 15 mm. The diode 60012 in radial orientation provides data closest to the surface, followed by the ion chamber.

Profiles within the central part of the 10×10 cm^2^ field in homogenous medium (Figure 3b) are flat within the field center and slightly decline towards the edge of the field. There are no obvious differences between the detectors (0.5%). In the same field, but with the cylinder inserted (Figure 3a), detector signals rise close to the cylinder surface. At 3 mm distance from the cylinder edge (x= 18 mm) the normalized signals increase between 1.0% and 2.5% depending on the detector. The increase is largest for the ion chamber and unshielded diode in radial orientation, followed by the microDiamond, the unshielded and finally the shielded diode, all in axial orientation.

For the 2×2 cm^2^ field in water only (Figure 4b), detector signals increase with reduced distance to the field edge, with a steep rise from a lateral position of approximately 25 mm towards positions closer to the field center. In a similar manner as in the 10×10 cm^2^ field with cylinder, the increase is largest for the ion chamber, followed by the unshielded diode in radial orientation and the microDiamond, the unshielded in axial orientation and finally the shielded diode. Profiles acquired with the inserted cylinder (Figure 4a) yield similar results.

Two observations can be made from the ratios of the 2×2 cm^2^ field (Figure 4c): First, the signal increase towards the field center is smaller for all detectors when the cylinder is inserted. Second, the signals are between 5% and 10% higher far outside the field at distances larger than 25 mm from the center when the cylinder is inserted. The increase is largest for the microDiamond and the ion chamber and the smallest for the shielded diode.

### 3.2 Calculations and film measurements

**FIGURE 5:**
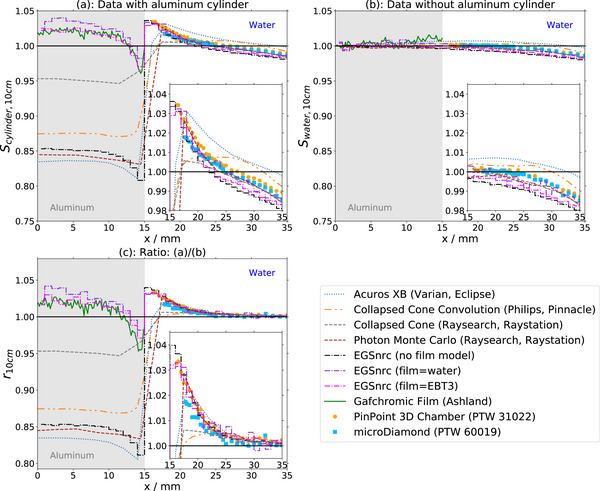
Ratios of dose profiles obtained with and without an aluminum cylinder in water using different dose calculation tools and film measurements for the field size 10×10 cm^2^. The cylinder center is located at position 0 mm, the cylinder edge at 15 mm. In the Monte Carlo simulation using EGSnrc dose to medium is reported. Three cases are distinguished: For the case called ‘EGSnrc (no film)’ the dose is scored in a 1 mm thick layer in the aluminum‐water‐phantom. For the case ‘EGSnrc (film = water)’ a 1 mm thick layer of water is placed in the middle of the cylinder as a first approximation of the film measurement. For the last case ‘EGSnrc (film = EBT3)’ dose is scored in a 28 µm thick active layer composed according to Palmer et al.^40^ sandwiched by two polyester layers with a thickness of 0.125 mm.

For the calculations as well as the film measurements, dose within the cylinder was also accessible (Figure 5/6). The main contributions to measurement uncertainty and their magnitude are summarized in Table 3. In the 10×10 cm^2^ field (Figure 5), only a small increase of 0.01 of the dose was obtained in film measurements in the central part of the cylinder, while the ratio decreased by up to 0.05 within the cylinder material close to the material interface at radial position of 15 mm. EGSnrc simulations yielded similar ratios inside the cylinder when modelling the dose scoring plane as either water or film material. A decline close to the cylinder wall was observed, although its position and magnitude differed by 1 mm and 0.2, respectively, from the form observed in the film measurements.

**TABLE 3:** Uncertainty analysis for the ratios $r_{\mathrm{FS}}\left(x\right)$ according to equation 2, obtained in the 2×2 cm^2^ field. Data are given exemplary for the diode in radial orientation positioned at the positions 17 mm and 25 mm (2 mm and 10 mm from the cylinder edge, respectively). The uncertainties related to position variations and material composition (marked with^a^), where estimated as uncertainties in dose using the treatment planning system.

	Estimated uncertainty [%]		
Uncertainty	17 mm	25 mm	Comment
***Type A***			
Detectors			
signal reproducibility/noise	0.50	0.49	RMSD (root‐mean‐square deviation) according to parabola fit ± 1 mm around points of interest
Film			
signal reproducibility/noise	3.2	6.4	averaging over 3 film measurements and 11 pixels perpendicular to profile direction
Monte Carlo calculations			
statistical uncertainty	0.14	0.12	depends on number of histories and radial distance (direct beam vs. only scattered radiation)
***Type B***			
Measurement setup			
alignment of cylinder in field center^a^	3.8	0.48	±0.5 mm, relevant for comparison between detectors
Detectors			
position of effective point of measurement^a^	2.6	0.97	relevant for radial orientation, ± 0.2 mm, estimated from^42^
Film			
alignment with respect to interface^a^	2.5	1.4	alignment of three measurements and measurements with and without aluminum. Deviation of one pixel: 0.17 mm
Geometry modeling			
cylinder density^a^	1.8	2.4	measurement material composition (density = 2.8 g/cm^3^) differs from simulation composition (density = 2.7 g/cm^3^)
TPS calculations			
dose grid resolution^a^:			resolution: 1 mm, 2 mm
‐ setup with cylinder	1.6	1.9	
‐ setup just water phantom	2.7	1.0	
***Total Uncertainties***			
for detectors	4.6	1.2	
for film	4.1	6.5	
for TPS	3.6	2.6	
for Monte Carlo	1.8	2.4	

In the 10 × 10 cm^2^ field without the cylinder, all studied dose engines provided similar data in the field center with minor differences towards the edge of the field (maximum difference 0.02 between Acuros XB and Photon Monte Carlo). In the 10×10 cm^2^ field within the aluminum cylinder, there are large differences between the algorithms (maximum difference 0.12 between Acuros and Raystation Collapsed Cone). The reported dose depends on the reporting mode (dose‐to‐water or dose‐to‐medium) and assigned medium (film modelled as water, film material or aluminum). In the vicinity of the cylinder, there are systematic differences between algorithms. Especially Acuros XB und Pinnacle Collapsed Cone deviate systematically from the other algorithms by up to 1.2% between Acuros/CCC and Raystation Monte Carlo for x‐positions larger than 25 mm. Dose obtained with film closely matches dose calculated by EGSnrc using a realistic film composition. There are no distinct differences between the simulations between using the realistic film composition and water instead of the film material.

**FIGURE 6:**
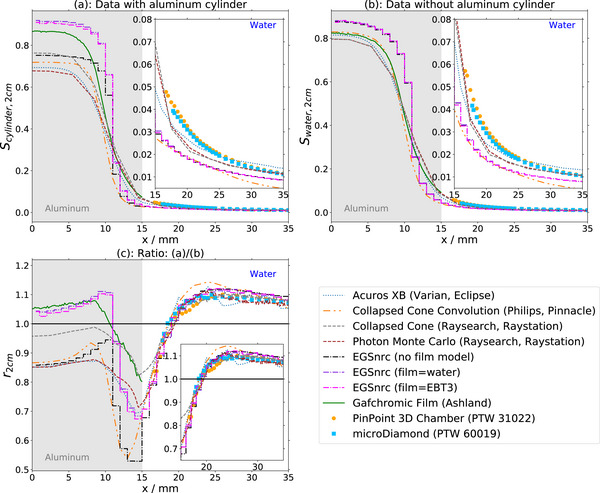
Ratios of dose profiles obtained with and without an aluminum cylinder in water using different dose calculation tools and film measurements for the field size 2×2 cm^2^. The cylinder center is located at position 0 mm, the cylinder edge at 15 mm. In the Monte Carlo simulation using EGSnrc dose to medium is reported. Three cases are distinguished: For the case called ‘EGSnrc (no film)’ the dose is scored in a 1 mm thick layer in the aluminum‐water‐phantom. For the case ‘EGSnrc (film = water)’ a 1 mm thick layer of water is placed in the middle of the cylinder as a first approximation of the film measurement. For the last case ‘EGSnrc (film = EBT3)’ dose is scored in a 28 µm thick active layer composed according to Palmer et al.^40^ sandwiched by two polyester layers with a thickness of 0.125 mm.

In the 2×2 cm^2^ field without the cylinder, doses by different engines vary by 0.035 in the field center. The dose profiles vary considerably at the field edge (x = 12 mm, maximum difference 0.15 between Pinnacle Collapsed Cone Convolution and Raystation Photon Monte Carlo). Differences further away from the field edge (x = 20 mm) are still 0.07. With the aluminum cylinder inserted, variation of dose in the field center calculated by different engines increases (0.1 maximum). Dose profiles outside of the cylinder are similar in shape. Differences are visible in the ratios (Figure 6c): From the field center towards the field edge, both in the film measurement and in the calculations, first an increase is observed, then a decrease still within the cylinder close to the interface, with the largest drop observed for the Monte Carlo simulations and Pinnacle Collapsed Cone Convolution. Outside the cylinder, dose ratios increase, reaching a ratio of 1.0 at 4 mm from the edge and a maximum 10 mm from the edge (*x* = 25 mm) Further away from the cylinder, ratios stay at a value between 1.07 and 1.09 for all algorithms.

## 4 DISCUSSION

### 4.1 Detector response in the vicinity of the aluminum cylinder

When a cylinder is entered in the water phantom and irradiated, a larger number of secondary electrons is released than in the setup with just water. Therefore, electrons, especially those generated close to the edge of the cylinder, scatter out of the cylinder and create an increase in dose within the distance they are transported. Electrons will interact with material of the detectors brought into the vicinity of the cylinder edge. At the same time, more photons will be scattered when the cylinder is inserted. Scattered photons will reach the detectors and interact with the materials even when placed further away from the field edge.

When inserting the aluminum cylinder in the 10×10 cm^2^ field, detector signals increase within around a centimeter from the cylinder edge. Detector signal differences are in line with the typical explanations of detector response differences: The shielded diode contains high density components that will filter out some of the electrons, resulting in a smaller increase in signal than for the other detectors. The ion chamber has less perturbating material close to the active volume and, therefore, detects a larger portion of the additional electrons. The higher response of the diode in radial compared to axial orientation is also consistent with the model: When used in the radial orientation, electrons need to travel a smaller lateral distance to reach the active area than in the axial orientation. Comparing the results of the detector specific Monte Carlo simulations in Table 4, the results show differences due to the different geometry of the beam, but as expected the relative order of the single detectors with respect to the response is the same as in the measurements and is consistent with the expectations of the model explained before. The similar relative behavior of the studied detector types has been observed by Wegener and Sauer^42^ in a setup blocking the central part of the beam, such that only scattered radiation reached the detectors.

**TABLE 4:** Results of Monte Carlo simulations for three types of detectors in a radial distance of 18 mm from the isocenter and a depth of 75 mm for two field sizes using the DosRZ Package of EGSnrc. Normalized detection volume dose for simulations with and without aluminum cylinder in the field are presented together with the ratio of both. All doses were normalized using the simulation for the 10 × 10 cm^2^ field without cylinder. The uncertainties are calculated from the statistical uncertainties of the simulation using Gaussian error propagation.

	2 × 2 cm^2^	10 × 10 cm^2^
**PTW‐Pinpoint 3D 31022**		
With cylinder	0.022 ± 0.001	1.026 ± 0.001
Without cylinder	0.026 ± 0.001	1.000 ± 0.001
Ratio	0.847 ± 0.002	1.026 ± 0.001
**PTW‐Diode 60012**		
With cylinder	0.022 ± 0.001	1.020 ± 0.002
Without cylinder	0.024 ± 0.001	1.000 ± 0.002
Ratio	0.943 ± 0.004	1.020 ± 0.002
**Water‐equivalent Detector**		
With cylinder	0.023 ± 0.001	1.028 ± 0.001
Without cylinder	0.027 ± 0.001	1.000 ± 0.001
Ratio	0.881 ± 0.001	1.028 ± 0.001

Due to the changing electron spectrum and volume effect with field size, output correction factors are necessary for small fields. For the 2 × 2 cm^2^ field, the interface is outside the primary beam and these factors are not applicable. It is known that high‐Z detector material does over‐respond outside the useful beam. This is due to the lower energy of the scatter photons. As we cross‐calibrated the detector responses far enough from the interface, this is not relevant for our data. In this configuration, a decrease of the measured signals is observed close to the field edge when the cylinder is inserted. As the field only irradiates the central part of the cylinder, all released secondary electrons need to travel at least 5 mm distance through aluminum to escape into water. Therefore, the number of electrons, and consequently the dose, in the vicinity of the cylinder declines. The drop in signal for the ion chamber is the largest, which is expected when we assume that this particular detector detected a larger proportion of electrons in the open field than the other detectors. The microDiamond and the PinPoint ion chamber respond very similar to each other. Volume averaging effects for the detectors are 2.2% for the case without cylinder in the field and 0.5%‐0.8% with cylinder in the field at a distance each of *x* = 18 mm Therefore, the volume averaging effect is only a minor contribution to the increased detector signal of the ion chamber. In the same way as explained for the 10×10cm^2^ field, the detector specific Monte Carlo simulations are also consistent with respect to the order of the single detectors for in the signal‐response for both measurements with and without cylinder in the field (compare Table 4).

Several centimeters away from the cylinder edge, changes in detector response cannot be attributed to electrons, but to scattered photons created in the presence of the aluminum cylinder. As expected, the signal of the shielded diode designed to compensate the signal in low‐energy scatter conditions, changes less than the signal of the unshielded diode. Based on these results, we recommend the use of at least two different detector types for measurements close to inhomogeneities. Air‐filled ionization chambers are the best option when volume effects are absent because of their low energy dependence. So we recommend a combination of a solid state detector and a small ionization chamber that is cross‐calibrated sufficiently away from the interface. The PinPoint ion chamber and microdiamond produced the most consistent results in this study.

One of the limitations of the study are the simplified detector models in the radial geometric setup including the omission or approximation of finer details around the detector active volume, such as the detector shells. In the future, a more detailed Monte Carlo study will be initiated with a more detailed modelling of the detectors. By tracking the interactions of the different particles a better understanding shall be obtained, which kind of particles originating from which region are blocked by the denser parts of detectors or reach the active volume of the different types of detectors.

Another limitation is the use of an aluminum cylinder. Instead of bone, bone‐equivalent synthetic materials^5^ or aluminum^43^ were used in similar setups. The use of aluminum as a substitute of bone was recently discussed in a study evaluating the performance of different dose engines^44^ Scatter effects are a little stronger than for bone. The density of aluminum, at 2.70 g/cm^3^, is higher than the density of bone, at 1.65 g/cm^3^, but in terms of effective atomic number the two materials show similar properties (Aluminum Z = 13, bone Z_eff _= 12.31)^45^

### 4.2 Dose calculation within the aluminum cylinder

Dose ratios reported within the central part of the aluminum heavily depend on the calculation algorithm and dose reporting mode (Figure 5/6). Even the more advanced type C algorithms (Raystation Monte Carlo, EGSnrc and Varian AcurosXB) vary on the central cylinder axis in terms of absolute dose. The reduction (Figure 5c and 6c) is similar, as expected from the attenuation coefficient^46^ of the materials for typical photon energies of a 6 MV beam after the incident beam has passed 1.5 cm of aluminum instead of 1.5 cm of water.

The Collapsed Cone algorithms implemented in two different planning systems yield dose ratios which are approximately five percentage points (Pinnacle) and ten percentage points (Raystation) higher than the ratios obtained with the three previously described algorithms. This illustrates why Monte Carlo or the AcurosXB algorithms should be preferred for dose calculations when the dose to bone is of interest, as already recommended in international guidelines.^47^

### 4.3 Dose calculation in the vicinity of the material interface

The rise in dose close to the interface in the large field (Figure 5) calculated by EGSnrc is comparable to the results obtained with film. It needs to be noted that, this calculation model used a simplified radially symmetric geometry including a circular field and a radiation source providing a parallel beam. All other calculation models fail to predict the increase. In general, the curves close to the interface look smoothed out for most calculation models, which is partly due to the choice of the calculation grid size. The estimated uncertainty (Table 3) does not fully account for the differences. Acuros XB and Raystation MC at least reproduce some of the increase, while all Collapsed Cone algorithms provide no indication of an increase. This is due to the fact, that CC uses energy deposition kernels obtained in water only and does not adopt the kernels to materials with different atomic numbers.^28^

In the smaller field (Figure 6) large differences between the algorithms are observed within the cylinder part that is not irradiated by the primary beam. Differences here are a combination of algorithm performance and details of the underlying treatment planning systems. This study illustrates that several independently commissioned dose engines yield completely different results especially at field edges but even within the center of open fields. It can be assumed that the differences are due to the commissioning process, for example due to the quality of entered beam profiles, field output factors or beam modelling. We therefore recommend extending beam model verification efforts towards using more complex inhomogeneities at different field sizes, matching what is expected to be encountered clinically. The variation between different clinically used algorithms illustrates that homogeneous phantoms for plan‐specific quality assurance can hardly be enough to validate beam models. In addition to controlled sample geometries (such as the cylinder), validation of dose calculation in inhomogeneous anthropomorphic phantoms is ultimately desired. Among the main challenges remains the choice of appropriate small size detectors and detector corrections necessary for the vast amount of possible measurement conditions, including aspects not yet discussed in this context, such as angular dependence.

When performing measurements near interfaces and a discrepancy between measured and calculated data is encountered, one needs to carefully examine the cause of the deviation, as this study illustrates that neither incorrect measurement data nor incorrect calculations can be excluded in such a scenario. In a clinical setting we recommend the use of a micro ionization chamber. To achieve data closer to a high density structure, a silicon or a diamond diode may be used. Cross calibration with the ion chamber and corroboration with a second detector will provide data with small errors plus an uncertainty estimation. Calculation data should be reproduced with a different algorithm and positioning should be verified, especially in high gradient regions.

## 5 CONCLUSIONS

Dose calculation in inhomogeneous geometries is challenging for many algorithms and a spread of results is observed within and in the vicinity of a larger density material inserted into water. Differences between the dose calculations of three commercial treatment planning systems are due to dose reporting media, beam data commissioning and also due to the physical models of the different algorithms. For the Collapsed Cone algorithms no backscattering is reproduced.

Detector response was studied in water around an aluminum cylinder, serving as a simple model for bone in tissue encountered in the body. The PinPoint ion chamber and the microDiamond consistently showed very similar response (within 3% in the case of the 2×2 cm^2^ field and 1% for the 10×10 cm^2^ field in a distance of *x* = 18 mm) in the vicinity of the aluminum cylinder. Their measurements were comparable to the data by most dose engines in the 10×10 cm^2^ field. However, measurement data suggested higher dose than calculated in the 2×2 cm^2^ field (0.005 at *x* = 20 mm).

One of the main limitations of currently available detectors are their outer dimensions, rendering measurements very close to the interface technically impossible. The PinPoint chamber and diode in radial orientation allowed the closest approach with a first measuring point just 0.6 mm or 1.45 mm from the interface. Positioned in an asymmetric way with respect to the field in radial orientation, the unshielded diode may lead to large discrepancies in other measurement conditions.

## AUTHOR CONTRIBUTIONS

Jonas Ringholz: Conceptualization, data acquisition, Monte Carlo simulation and dose engine calculations, analysis, discussion and interpretation of the data, manuscript initial draft preparation. Otto Andreas Sauer: Concept design, discussion and interpretation of the data, manuscript critical review and editing, acquisition of funding. Sonja Wegener: Concept design, discussion and interpretation of the data, manuscript critical review and editing.

## CONFLICT OF INTEREST STATEMENT

There are no conflicts of interest to disclose.
